# Flavoprotein fluorescence elevation is a marker of mitochondrial oxidative stress in patients with retinal disease

**DOI:** 10.3389/fopht.2023.1110501

**Published:** 2023-02-16

**Authors:** Sofia Ahsanuddin, Hernan A. Rios, Oscar Otero-Marquez, Jason Macanian, Davis Zhou, Collin Rich, Richard B. Rosen

**Affiliations:** ^1^ Department of Ophthalmology, New York Eye and Ear Infirmary of Mount Sinai, New York, NY, United States; ^2^ Department of Ophthalmology, Icahn School of Medicine at Mount Sinai, New York, NY, United States; ^3^ Department of Medical Education, New York Medical College, Valhalla, NY, United States; ^4^ OcuSciences Inc., Ann Arbor, MI, United States

**Keywords:** retina, oxidative stress, mitochondrial dysfunction, flavoprotein fluorescence imaging, diabetic retinopathy, central serous chorio retinopathy (CSCR), exudative age related macular degeneration, retinal vein occlusion (RVO)

## Abstract

**Purpose:**

Recent studies of glaucoma, age-related macular degeneration, and diabetic retinopathy have demonstrated that flavoprotein fluorescence (FPF) can be utilized non-invasively as an indicator of mitochondrial oxidative stress in the retina. However, a comprehensive assessment of the validity and reliability of FPF in differentiating between healthy and diseased eyes across multiple disease states is lacking. Here, we evaluate the sensitivity and specificity of FPF in discriminating between healthy and diseased eyes in four leading causes of visual impairment worldwide, one of which has not been previously evaluated using FPF. We also evaluate the association between FPF and visual acuity.

**Methods:**

A total of 88 eyes [21 eyes of 21 unaffected controls, 20 eyes from 20 retinal vein occlusion (RVO) patients, 20 eyes from 20 diabetic retinopathy (DR) patients, 17 eyes from 17 chronic exudative age-related macular degeneration (exudative AMD) patients, and 10 eyes from 10 central serous retinopathy (CSR) patients] were included in the present cross-sectional observational study. Eyes were imaged non-invasively using a specially configured fundus camera OcuMet Beacon^®^ (OcuSciences, Ann Arbor, MI). The macula was illuminated using a narrow bandwidth blue light (455 – 470 nm) and fluorescence was recorded using a narrow notch filter to match the peak emission of flavoproteins from 520 to 540 nm. AUROC analysis was used to determine the sensitivity of FPF in discriminating between diseased eyes and healthy eyes. Nonparametric Kruskal-Wallis Tests with *post-hoc* Mann Whitney U tests with the Holm-Bonferroni correction were performed to assess differences in FPF intensity, FPF heterogeneity, and best corrected visual acuity (BCVA) between the five groups. Spearman rank correlation coefficients were calculated to assess the relationship between FPF and BCVA.

**Results:**

AUROC analysis indicated that FPF intensity is highly sensitive for detecting disease, particularly for exudative AMD subjects (0.989; 95% CI = 0.963 – 1.000, *p*=3.0 x 10^7^). A significant difference was detected between the FPF intensity, FPF heterogeneity, and BCVA in all four disease states compared to unaffected controls (Kruskal-Wallis Tests, *p* = 1.06 x 10^-8^, *p* = 0.002, *p* = 5.54 x 10^-8^, respectively). Compared to healthy controls, FPF intensity values were significantly higher in RVO, DR, exudative AMD, and CSR (*p* < 0.001*, p* < 0.001*, p* < 0.001, and *p* = 0.001, respectively). Spearman rank correlation coefficient between FPF intensity and BCVA was ρ *= 0.595* (*p* = 9.62 x 10^-10^).

**Conclusions:**

Despite variations in structural retinal findings, FPF was found to be highly sensitive for detecting retinal disease. Significant FPF elevation were seen in all four disease states, with the exudative AMD patients exhibiting the highest FPF values compared to DR, CSR, and RVO subjects. This is consistent with the hypothesis that there is elevated oxidative stress in all of these conditions as previously demonstrated by blood studies. FPF intensity is moderately correlated with the late-in disease-marker BCVA, which suggests that the degree of FPF elevation can be used as a metabolic indicator of disease severity.

## Introduction

Mitochondria are the essential cellular components for energy production, apoptosis, steroid synthesis, cellular signaling, and maintenance of homeostasis (1–4). They are the site of oxidative phosphorylation and are thus vital for oxygen-dependent adenosine triphosphate (ATP) production. Retinal cells are highly metabolically active with high baseline oxygen consumption demands and are thus particularly susceptible to mitochondrial dysfunction. Apoptosis may occur through the release of reactive oxygen metabolites that impair mitochondrial DNA (mtDNA) and the electron transport chain (5, 6). Inappropriate induction of apoptosis vis-à-vis activation of caspase-3 results in premature loss of cells and subsequent tissue dysfunction (7).

Mitochondrial dysfunction secondary to oxidative stress has been demonstrated to drive the pathogenesis of numerous retinal diseases such as age-related macular degeneration (AMD), diabetic retinopathy (DR), and retinal vein occlusion (RVO) (8–11). Oxidative stress results from the accumulation of reactive oxygen species that exceeds the antioxidant defense capacity of the cell (12). In healthy cells, reactive oxygen species are scavenged by antioxidant enzymes like manganese superoxide dismutase (5). Under stress, flavoproteins linked to mitochondrial enzymes in the electron transport chain become oxidized and emit a green autofluorescence when stimulated by blue light (13–16). The spectral properties of flavoproteins have previously been demonstrated to be distinct from those of other retinal fluorophores, such as lipofuscin (4). Thus, assessing levels of mitochondrial flavoprotein fluorescence (FPF) can potentially serve as a measure of metabolic dysfunction present within the human retina (4, 17).

Traditionally, retinal damage has been assessed by structural changes that occur in the setting of disease. However, this approach shows the end-result of metabolic disturbance but is not sensitive to detecting real-time fluctuations in oxidative stress. Recently, a method of functional imaging of mitochondria has been developed which provides *in vivo* quantitative information of FPF. Prior studies involving dry age-related macular degeneration, pseudotumor cerebri, glaucoma, and central serous retinopathy measured FPF by imaging patients’ retinas with a back-illuminated electron-multiplying charged-coupled device (EMCCD) camera equipped with a 512 x 512 pixel chip (14, 15, 18–22). These retinal metabolic imaging devices have undergone three iterations, with the first iteration capturing FPF over a 3° field of view (4). The latest generation of the device captures infrared images of 60° x 21.5° and an FPF image of 17° x 21.5° field of view. All three generations use a 1-ms flash of blue light (455 – 470 nm) to capture fluorescence from 520 - 540 nm. FPF is primarily expressed as two parameters known as FPF intensity and FPF heterogeneity (4). The former reflects global signal strength by measuring average pixel intensity over a 5.5 mm-diameter region centered at either the macula or the optic nerve head (4, 23). The latter captures the variation of signal intensity of all the pixels in the region of interest in the imaged eye. Both elevated levels of FPF intensity and heterogeneity have been correlated with disease progression and may be of value for evaluation of clinical severity, acuity of disease, and predicting prognosis (21, 24, 25).

Building upon these previous works, the present study uses the OcuMet Beacon^®^, a third-generation retinal metabolic analysis device, to quantify FPF in patients with four leading causes of visual impairment (26). One of the main objectives of this study was to evaluate the sensitivity and specificity of the OcuMet Beacon^®^ in discriminating between healthy control eyes and diseased eyes. A second objective was to expand the clinical investigation of FPF to subjects with retinal vein occlusion (RVO), a condition that has not been studied to date. Finally, our third objective was to investigate the association between FPF and best corrected visual acuity (BCVA) across different disease states. Here, we demonstrate the consistent response of FPF as a sensitive indicator of metabolic dysfunction across a variety of disease states and its significant association with visual acuity. With further characterization, FPF could become a clinically useful means of assessing therapeutic impact of a variety of interventions aimed at reducing oxidative stress in the human retina.

## Materials and methods

### Study population

This cross-sectional observational study was conducted at New York Eye and Ear Infirmary of Mount Sinai in New York, USA. The study protocol was approved by the Institutional Review Board (IRB) and adhered to the guidelines outlined in the Declaration of Helsinki. Written informed consent was obtained from all participants prior to their inclusion in the study and involved discussion of the study methodology along with associated risks and benefits. A total of 76 subjects and 24 age-matched healthy controls were recruited between October 2021 and August 2022. Patients’ demographic information, comorbidity data, chronology of illness, retinal diagnosis, and sub-classification of their retinal disease was collated through review of their electronic medical records and confirmed during the consent process. Three controls were excluded from the present study because it was discovered after recruitment that they were diagnosed with Type 2 diabetes mellitus. One control, two RVO, and three DR subjects were excluded after data collection because it was discovered during retrospective chart review that the patients had a prior history of cataract surgery in the study eye. One subject with central retinal vein occlusion (CRVO) was excluded because he was diagnosed concurrently with glaucoma. One subject’s data with central serous retinopathy (CSR) was excluded because the patient reported a diagnosis of sarcoidosis after enrollment. Two subjects with exudative AMD were excluded because of poor image quality secondary to improper positioning of the patient’s head on the chin rest.

The final analysis cohort included images from 67 subjects and 21 age-matched healthy controls. Inclusion criteria for the study subjects were the singular diagnoses of RVO, DR with or without macular edema, exudative AMD, or CSR in the imaged eye, in addition to natural crystalline lens, good fixation, clear media, and a BCVA better than 20/200 in Snellen. Subjects with other concomitant retinal pathologies in addition to the primary diagnosis were excluded from the study. Other exclusion criteria included history of prior vitreoretinal surgery or surgery impacting the corneal curvature; history of laser surgery within the past 3 months; history of cataract surgery due to data demonstrating that artificial intraocular lens exhibit increased levels of fluorescence compared to natural crystalline lens; uncontrolled hypertension; the presence of systemic vascular disease such as sickle cell disease and systemic inflammatory diseases like human immunodeficiency virus (HIV); current systemic immunosuppression; diabetes mellitus; retinal or vitreous hemorrhages that obscure the macula; ≥ Grade 3 nuclear sclerotic, cortical, or posterior subcapsular cataract based on the Lens Opacity Classification System III; inability to fixate; inability to comply with study procedures; active anterior chamber inflammation; and neurodegenerative disorders such as Alzheimer’s and Parkinson’s disease. Only one eye from each subject was included for imaging and data analysis to avoid interocular correlation. Enrollment of age-matched controls involved recruiting subjects between 43 and 74 years of age with natural crystalline lens with no evidence of vitreoretinal disease. Controls were also required to have a BCVA of 20/25 or better and no prior history of ocular pathology or ocular surgery.

Prior to enrollment in the study, all patients underwent a comprehensive ophthalmic evaluation including ETDRS visual acuity testing, slit lamp biomicroscopy, pupillary dilation using tropicamide 1% eye drops and phenylephrine hydrochloride 2.5% eye drops administered twice at 5-minute intervals, tono-pen tonometry (Reichert Technologies, Depew, NY), dilated fundus photography (Optos, Marlborough, MA), and OCTA imaging (XR Avanti; Optovue, Inc., Fremont, CA, USA). For patients in whom both eyes met the inclusion criteria, a single eye was selected at random for inclusion in the study.

Patients with DR were evaluated by a member of our retina service as non-proliferative diabetic retinopathy (NPDR) or proliferative diabetic retinopathy (PDR) with or without clinically significant macular edema according to the Early Treatment Diabetic Retinopathy Study (ETDRS) criteria (27). Patients with RVO were classified as possessing either central retinal vein occlusion (CRVO) or branch retinal vein occlusion (BRVO) through chart review and dilated fundus examination findings, color fundoscopic images, and IVFA images within six months of image acquisition. Patients with CSR were classified as active or chronic inactive using diagnostic wide-field IVFA imaging and OCT (28–30). Patients with exudative AMD were all noted to be advanced cases involving neovascularization as per the Age-Related Eye Disease Study (AREDS) Risk Factor Scoring System (31).

### Retinal metabolic analysis image acquisition and processing

**Figure 1:**
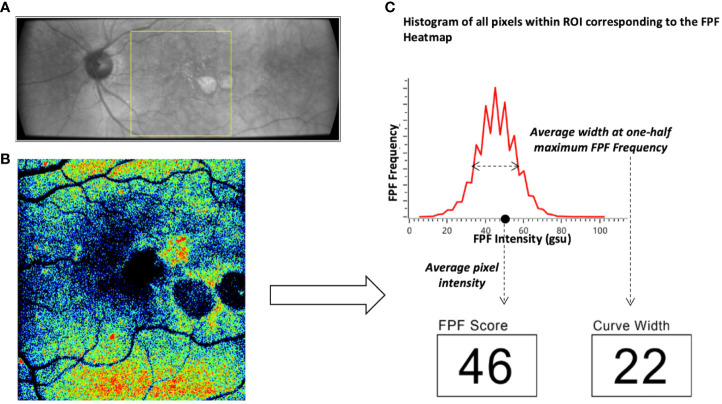
Overview of the OcuMet Beacon® imaging technique. **(A)** 60° x 21.5° infrared image with the region-of-interest (ROI) marked in yellow in a subject with exudative AMD. **(B)** Corresponding 17° x 21.5° FPF heatmap highlighting areas of increased mitochondrial dysfunction in warmer hues. **(C)** OcuMet Beacon's proprietary software automatically calculates the FPF intensity and heterogeneity using the pixel count. FPF intensity reflects the average pixel intensity over a 17° x 21.5° region centered at the macula. FPF heterogeneity captures the varation of signal intensity of all the pixels in the ROI.

**Figure 2:**
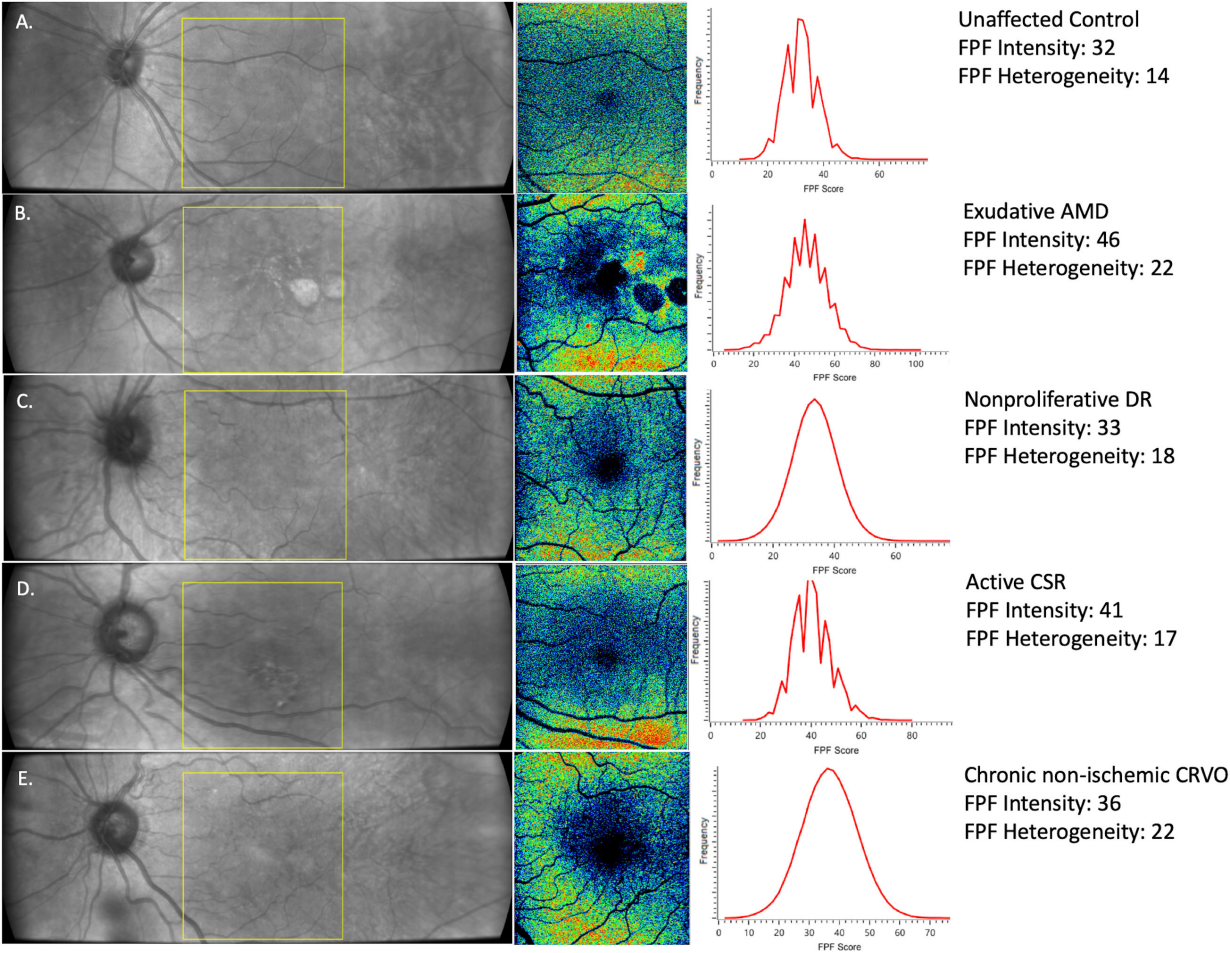
Representative images of **(A)** healthy control, **(B)** exudative AMD **(C)** nonproliferative DR, **(D)** active CSR, and **(E)** chronic non-ischemic CRVO. (Left) Infrared images of 60° x 21.5° with the yellow box indicating the region of interest (ROI) at the macula for FPF measurements. (Center) 17° x 21.5° FPF heat map. Warmer colors indicate higher FPF intensities. FPF heterogeneity is the average width of the histogram curve at one-half the maximum FPF frequency. (Right) Distribution of FPF intensities among all image pixels.

Functional imaging of retinal mitochondria was performed by measuring the FPF values at the macula using the OcuMet Beacon^®^ (OcuSciences, Ann Arbor, MI; Investigational Device) **(** **Figure 1** **)**. The device is classified as a Group 1 for light safety under American National Standards Institute (ANSI) Z80.36-2016 and International Organization for Standardization (ISO) 15004 guidelines. It captures infrared images of 60° x 21.5° and an FPF image of 17° x 21.5°. Optical filters for the excitation and peak flavoprotein emission are designed to maximize signal-to-noise ratio and minimize confounding fluorescence from other retinal fluorophores including lipofuscin. Each subject’s pupil was dilated prior to imaging. The macula was exposed to 60 ms of excitation flash. Images were excluded from the present study if they were of low quality due to poor focus, eyelash interference or pupil cropping. All images were stored as 1920 x 700 pixel 12-bit grayscale PNG files. The integrated Ocumet^®^ Image software package analyzed the images for average FPF pixel intensity and heterogeneity, from all pixels within a 17° rectangular field. One macular image per subject was included in the analysis and was subjectively evaluated by the same operator for image quality. FPF heterogeneity was also automatically calculated as the average width of the histogram curve at one-half the maximum FPF frequency (4). A composite of FPF and infrared images comparing a control subject’s retina to subjects with each of the disease states investigated in this study are found in **Figure 2**.

### Statistical analysis

Statistical analyses were performed using SPSS 24.0 (IBM Corporation, Chicago, IL, USA) and Microsoft Excel (Microsoft Corporation, Redmond, WA). BCVA in Snellen was converted to LogMAR for statistical analysis. A one-way ANOVA test with *post hoc* Tukey test was used to assess for any significant differences in age between the different disease groups compared to controls. Since there were limited sample sizes and not all groups met the Kolmogorov-Smirnov test for normality, initial analysis consisted of nonparametric Kruskal-Wallis Tests and the *post hoc* Mann-Whitney U tests with the Holm-Bonferroni correction for multiple comparisons. The *post hoc* analysis was intended to control the experiment-wise type-1 error rate.

Following stratification of the RVO, DR, and CSR disease groups, sub-analyses compared values in PDR and NPDR, active and chronic inactive CSR, and CRVO and BRVO patients to age-matched healthy controls, respectively. Correlation analyses were performed using Spearman’s rank correlation coefficient (ρ) because the FPF and BCVA data were not normally distributed. Univariate regression analysis was performed to evaluate the impact of FPF intensity and heterogeneity on BCVA. Area under the receiver operating characteristic (AUROC) curve with 95% confidence interval (CI) was used to assess the diagnostic ability of FPF intensity and FPF heterogeneity metrics to differentiate between healthy age-matched eyes (controls) and eyes with retinal pathology. AUROC curves were visualized using GraphPad Prism Version 9.0 (GraphPad Software, Inc., San Diego, CA). A *p* value less than 0.05 was considered statistically significant.

Intra-visit repeatability of OcuMet Beacon^®^ scans at the macula was characterized using the coefficient of variation. Four sequential images taken from a representative diseased eye and from a representative control eye were used to calculate this metric. The calculations were derived from the standard deviation of obtained FPF values divided by their mean.

## Results

### Participant characteristics

Demographic characteristics for all subjects and controls are included in **Supplementary Table 1**. 67 out of 76 subjects referred for retinal metabolic imaging met the inclusion criteria and were imaged using the OcuMet Beacon^®^ to determine the average FPF pixel intensity for the imaged eye as well as their BCVA in Snellen at their last visit. Overall, the median age ± interquartile range (IQR) was 58.5 ± 13 years and 42 (47.7%) were female. Race distribution was 40 (45.4%) White, 17 (19.3%) Black, 8 (9.1%) Asian, and 23 (26.1%) Hispanic. Eye laterality was 43 (48.9%) OD and 45 (51.1%) OS. The median age ± IQR for healthy control subjects was 55 ± 9 (*N* = 21, range: 43 - 74 years) compared to 59 ± 10 for the RVO cohort (*N* = 20, range: 36 - 77 years), 59 ± 11.25 for the DR cohort (*N* = 20, range: 40 - 80 years), 70 ± 15 for the exudative AMD cohort (*N* = 17, range: 48 - 87 years), and 54 ± 11.75 for the CSR cohort (*N* = 10, range: 35 - 65 years).

### Intra-visit repeatability

The coefficients of variation for FPF intensity and FPF heterogeneity in a representative diseased eye were 1.68% and 16.39%, respectively. For an unaffected control eye, the coefficients of variation for FPF intensity and FPF heterogeneity were 2.82% and 7.29%, respectively.

### Area under the receiver operating characteristic analyses

**Figure 3:**
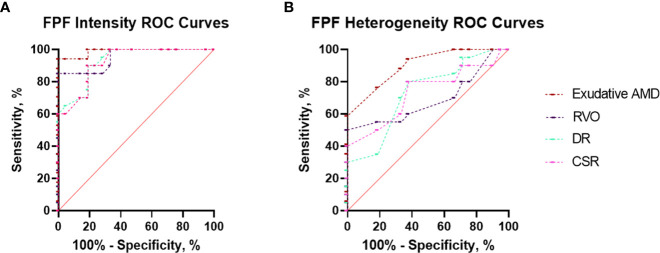
Area under the receiver operating characteristic (AUROC) curve analyses demonstrating the diagnostic ability of **(A)** FPF intensity and **(B)** heterogeneity to discriminate between eyes with retinal pathology (exudative AMD, RVO, DR, and CSR) and unaffected control eyes.

AUROC analysis was utilized to compare the ability of flavoprotein fluorescence metrics to differentiate patients with all four disease states from unaffected controls. The results of the AUROC curve analyses are shown in **Figure 3** and **Table 1**. FPF intensity displayed consistent diagnostic capability when differentiating between healthy age-matched eyes and diseased eyes at the region of interest (ROI), with AUROC values ranging from 0.927, 0.951, 0.989, and 0.921 for RVO, DR, exudative AMD, and CSR, respectively. FPF heterogeneity demonstrated poor diagnostic capability for RVO (AUROC = 0.689). Good diagnostic capability was demonstrated for DR and CSR (AUROC = 0.730 and 0.731, respectively). Highest differentiation was noted for FPF heterogeneity in terms of exudative AMD (AUROC = 0.901).

**Table 1:** Area under the receiver operating curve (AUROC) analysis for differentiating healthy eyes (controls) and eyes with retinal pathology based on FPF intensity and heterogeneity.

Measurements		AUROC (95% CI)	Std. Error	*P* Value
FPF Intensity				
	**Controls *vs*. RVO**	0.927 (0.853 – 1.000)	0.038	3.0 x 10^-6^*
	**Controls *vs*. DR**	0.951 (0.890 – 1.000)	0.031	7.7 x 10^-7^*
	**Controls *vs*. Exudative AMD**	0.989 (0.963 – 1.000)	0.013	3.0 x 10^-7^*
	**Controls *vs*. CSR**	0.921 (0.828 – 1.000)	0.047	1.8 x 10^-4^*
FPF Heterogeneity				
	**Controls *vs*. RVO**	0.689 (0.518 – 0.861)	0.088	0.038*
	**Controls *vs*. DR**	0.730 (0.575 – 0.884)	0.079	0.012*
	**Controls *vs*. Exudative AMD**	0.901 (0.806 – 0.995)	0.048	2.7 x 10^-5^*
	**Controls *vs*. CSR**	0.731 (0.521 – 0.941)	0.107	0.040*

FPF intensity showed the largest AUROC of 0.989 for exudative AMD subjects, followed by DR, RVO, and CSR subjects. FPF heterogeneity had lower AUROC values ranging from 0.689 for RVO subjects and 0.901 for exudative AMD subjects. CI, confidence interval; RVO, retinal vein occlusion; DR, diabetic retinopathy; Exudative AMD, age-related macular degeneration; CSR, central serous retinopathy. *p* values < 0.05 is considered statistically significant.

The bolded values are statistically significant P values.

### Flavoprotein fluorescence analyses

**Figure 4:**
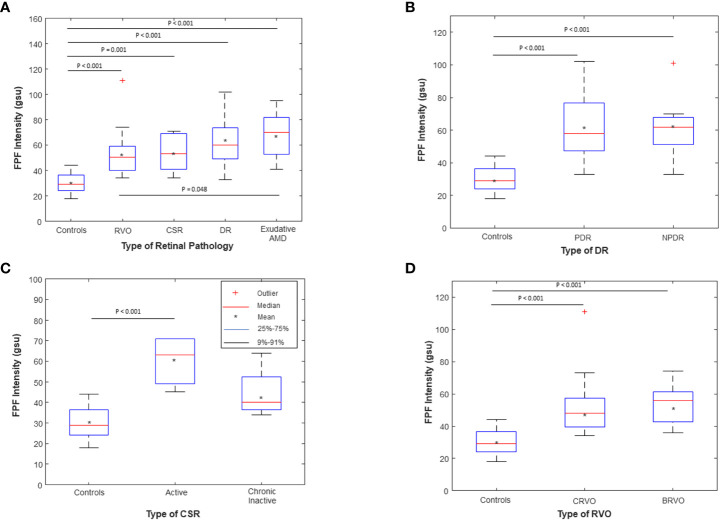
Box and whisker plots of flavoprotein fluorescence (FPF) average pixel intensity between **(A)** unaffected age-matched control subjects compared to RVO, CSR, DR, and Exudative AMD subjects; **(B)** Age-matched controls compared to PDR (N=11) and NPDR (N=9) subjects; **(C)** Age-matched controls compared to active (N=6) and chronic inactive (N=4) CSR subjects; and **(D)** Age-matched controls compared to CRVO (N=11) and BRVO (N=9) subjects. The means are indicated by crosses, medians by the horizontal lines, 25-75% quartiles by the boxes, and 9%-91% ranges by the whiskers. Significant *p* values for the *post hoc* pairwise comparisons after nonparametric Kruskal Wallis tests are shown; all other pairwise comparisons were not statistically significant (*p* > 0.05). RVO, retinal vein occlusion; DR, diabetic retinopathy; wet AMD, wet age-related macular degeneration; CSR, central serous retinopathy; PDR, proliferative diabetic retinopathy; NPDR, non-proliferative diabetic retinopathy; CRVO, central retinal vein occlusion; BRVO, branch retinal vein occlusion. The symbol * stands for the mean, indicated by the legend in Figure 4.

As shown in **Supplementary Table 2**, a Kruskal-Wallis Test demonstrated that there was a statistically significant difference in mean FPF intensity between the different disease groups compared to healthy controls (χ^2^(2) = 42.94, *p* = 1.06 x 10^-8^). Mean FPF (± SD) intensity values were lowest in the healthy control group (30.62 ± 8.03, 95% CI = 26.96 – 34.28), followed by RVO (53.80 ± 17.97, 95% CI = 45.39 – 62.21), CSR (53.80 ± 14.34, 95% CI = 43.54 – 64.06), and DR (61.75 ± 19.84, 95% CI = 52.47 – 71.03). Notably, the exudative AMD subjects had a mean FPF intensity score that was 2.2 times as large as the healthy control group (67.47 ± 17.77, 95% CI = 58.33 – 76.61). Box and whisker plots of FPF intensity values for each group are shown in **Figure 4A**. *Post hoc* pairwise comparisons after nonparametric Kruskal-Wallis tests indicated that FPF intensity values were significantly higher across all disease groups compared with the healthy control group (**Table S3** **)**. Importantly, exudative AMD subjects had significantly higher FPF intensity scores compared to RVO subjects (*p* = 0.048), indicating limited specificity of FPF for discriminating between different disease states **(** **Figure 4A** **)**.

Sub-analysis comparing PDR and NPDR subjects indicated that both groups had statistically significantly higher FPF intensity values compared to healthy controls (*p* < 0.001, *p* < 0.001, respectively, **Figure 4B**; **Table S4**). There was no statistically significant difference in FPF intensity between PDR and NPDR subjects (*p* = 0.948). Compared to controls, active CSR subjects had higher mean FPF intensity values (*p* < 0.001, **Figure 4C**; **Table S5**). There was no significant difference between chronic inactive CSR subjects and controls (*p* = 0.074). Both CRVO and BRVO subjects displayed statistically significantly higher FPF intensity values compared to controls (*p* < 0.001 and p < 0.001, respectively, **Figure 4D**; **Table S6**). There was no significant difference in FPF intensity scores between CRVO and BRVO subjects (*p* = 0.619).

FPF heterogeneity was statistically significant across all groups (χ^2^(2) = 17.327, *p* = 0.002) **(** **Supplementary Figure 1A**; **Table S2** **)**. Mean FPF heterogeneity (± SD) values of healthy controls, RVO, DR, exudative AMD, and CSR patients were 15.62 ± 2.87 (95% CI = 14.31 - 16.93), 18.00 ± 4.10 (95% CI = 16.08 - 19.92), 21.80 ± 10.44 (95% CI = 16.91 - 26.69), 23.12 ± 9.91 (95% CI = 18.02 - 28.21), 18.70 ± 4.19 (95% CI = 15.70 - 21.70), respectively. *Post-hoc* pairwise comparisons revealed that the difference between healthy controls and all four disease groups were statistically significant (**Supplementary Figure 1A**; **Table S3**). All other comparisons were not significant. Sub-analysis comparing PDR and NPDR subjects indicated that FPF heterogeneity was significantly elevated in both PDR and NPDR subjects compared to age-matched controls (*p* = 0.046 and *p* = 0.031, respectively, **Supplementary Figure 1B**; **Table S4**). FPF heterogeneity values were not statistically significantly different between active and chronic inactive CSR subjects compared to healthy controls (*p* = 0.117, **Supplementary Figure 1C**). FPF heterogeneity was also found to not be significantly different between CRVO, BRVO, and age-matched controls (*p* = 0.112, **Supplementary Figure 1D**).

**Figure 5:**
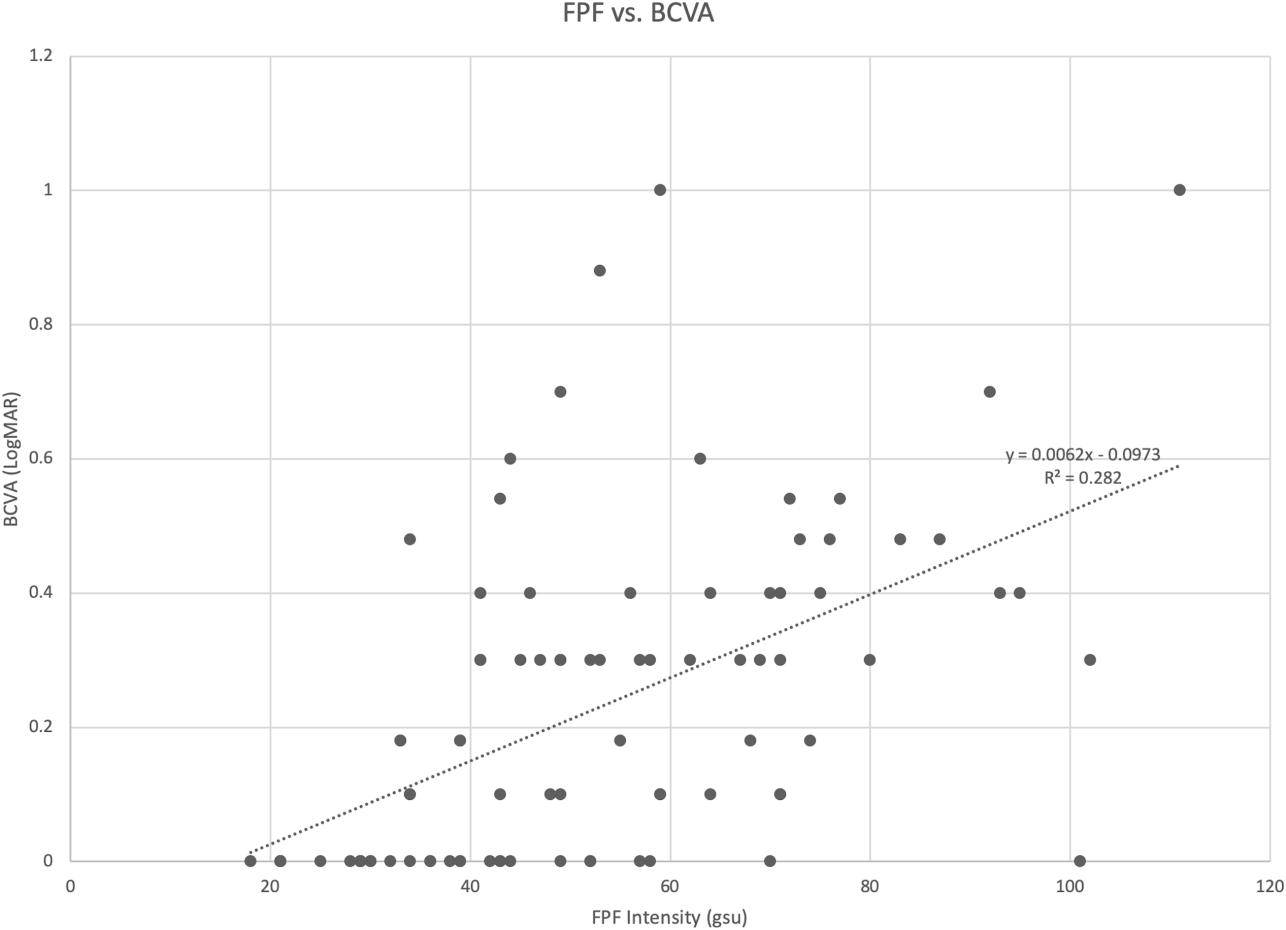
Scatter diagram illustrating the association between flavoprotein fluorescence (FPF) intensity and best corrected visual acuity (BCVA). The spearman rank correlation coefficient was 0.595 (95% CI = 0.362 – 0.666, *p* = 9.62 x 10^-10^).

Spearman rank correlation analysis revealed that FPF intensity values were moderately correlated with BCVA (ρ = 0.595, *p* = 9.62 x 10^-10^, 95% CI = 0.362 - 0.666) **(** **Table 2**; **Figure 5** **)**. FPF heterogeneity values were also found to be weakly correlated with BCVA (ρ = 0.306, *p* = 0.004, 95% CI = -0.089 - 0.323).

**Table 2:** Spearman rank correlation coefficients with 95% CI between FPF intensity and heterogeneity and BCVA.

FPF Intensity				
Clinical Characteristic*	Spearman Coefficient	5% CI Lower Limit	95% CI Upper Limit	*P* Value
BCVA	0.595	0.362	0.666	9.62 x 10^-10^*
FPF Heterogeneity				
Clinical Characteristic*	**Coefficient**	**5% CI Lower Limit**	**95% CI Upper Limit**	***P* Value**
BCVA	0.306	-0.089	0.323	0.004*

BCVA, Best Corrected Visual Acuity in LogMAR; CI, Confidence Interval. *Indicates statistical significance.

The bolded values are statistically significant P values.

### Best corrected visual acuity analyses

Best corrected visual acuity (BCVA) differences from controls was statistically significant across all groups (χ^2^(2) = 39.481, *p* = 5.54 x 10^-8^, **Supplementary Figure 2A**; **Table S2**). Mean BCVA (± SD) in LogMAR of healthy controls, RVO, DR, exudative AMD, and CSR patients were 0.00 ± 0.00, 0.38 ± 0.32 (95% CI = 0.23 - 0.53), 0.25 ± 0.18 (95% CI = 0.17 - 0.34), 0.32 ± 0.19 (95% CI = 0.23 - 0.42), and 0.18 ± 0.15 (95% CI = 0.06 - 0.29), respectively. *Post-hoc* pairwise comparisons revealed that the BCVA was significantly poorer in all four disease groups compared to healthy controls (**Table S3**). All other comparisons were not statistically significant. *Post-hoc* pairwise comparisons revealed that the BCVA of PDR and NPDR subjects were statistically significantly lower from those of age-matched controls (*p* < 0.001 and *p* = 0.001, respectively, **Supplementary Figure 2B**; **Table S4**). The BCVA between PDR and NPDR subjects were not significantly different (*p* = 0.274). The BCVA of both active and chronic inactive CSR subjects were significantly lower than that of age-matched controls (*p* < 0.001 and *p* = 0.002, respectively) (**Supplementary Figure 2C**; **Table S5** **)**. The BCVA of both CRVO and BRVO subjects were significantly lower than that of age-matched controls (*P* < 0.001 and *P* < 0.001, respectively, **Supplementary Figure 2D**; **Table S6**). There was no statistically significant difference between the BCVA of CRVO and BRVO subjects (*p* = 0.708).

## Discussion

Quantification of flavoprotein fluorescence (FPF) is a novel and potentially useful technology for assessing clinical severity, predicting prognosis, and detecting treatment response in patients suffering from a variety of retinal diseases. In this study, we chose to investigate retinal FPF in patients affected by the four leading pathologies affecting the macula of the eye: exudative AMD, DR, RVO, and CSR (26). Each of these disorders is the result of and/or cause of significant retinal oxidative stress. We sought to measure the clinical utility of FPF by determining its specificity and sensitivity in discriminating between healthy and diseased eyes. Additionally, while typically a later indicator of disease severity, BCVA is the key functional marker of retinal health. For this reason, we wanted to investigate the relationship between FPF and visual outcomes as measured by BCVA.

Unlike conventional structural tests such IV fluorescein angiography (IVFA) or dilated fundus photography, FPF non-invasively assesses mitochondrial dysfunction in the human retina *in vivo*. Mitochondria are most densely concentrated in the retinal nerve fiber layer, retinal ganglion cells, inner plexiform layer, outer plexiform layer, the outermost portion of photoreceptors, and the basal surface of RPE cells as demonstrated by cytochrome oxidase (COX) staining (32). As described by Chen et al. and Andrade Romo et al., FPF signal intensity directly corresponds to FAD^+^ molecules that are oxidized secondary to aging or disease (4, 21, 33). It cannot be elicited from cells that are already dead or in the final stages of apoptosis. The fluorescence signal from oxidized flavoproteins is distinct from conventional fundus autofluoresence (FAF) which is principally due to lipofuscin. The current generation of the OcuMet Beacon^®^ utilized for this study minimizes the contribution of fluorescence from retinal fluorophores like lipofuscin by using narrow filter bands for FPF excitation and emission peaks. The proposed mechanism by which FPF intensity correlates with mitochondrial dysfunction is that the latter results in increased turnover of lipid-rich cellular membranes, which in turn results in increased levels of bioactive lipids, reactive oxygen species, and lipid peroxides that upregulate NK-kB and p38 mitogen-activated protein kinases (MAPK) (34–36). The p38 MAPK pathway has been noted to be particularly active in the RPE and the neurosensory retina and has been implicated in the induction of proinflammatory cytokines such as IL-1*B*, TNF- **α**, and IL-6 (37, 38).

This is of particular importance given the fact that in our study, the subjects with the most elevated FPF intensity and heterogeneity scores were found within the exudative AMD cohort. As stated previously, the pathophysiology of exudative AMD involves degeneration of RPE cells secondary to the accumulation of ROS and subsequent oxidative stress damaging mtDNA in the RPE (6, 32). In health, the RPE plays a crucial role in maintaining retinal health by transporting blood-borne nutrients from the vasculature to photoreceptors and phagocytosing outer segments of photoreceptors (39). In exudative AMD, one theory proposes that impaired autophagy of outer segments of photoreceptors by the RPE contributes to the progression of disease (40). RPE injury is thought to provoke a chronic pro-inflammatory response in the Bruch membrane and the choroid (41). Development of an abnormal extracellular matrix precipitates further RPE damage, which subsequently leads to retinal atrophy and neovascularization of the choriocapillaris and choroid (42). It has been shown that human RPE cells treated with hydrogen peroxide demonstrate preferential damage to mtDNA due to oxidative stress *(* 43–45). Brown et al. have also demonstrated that RPE and photoreceptor damage in *Sod2* knock-out mice reduced RPE function and increased levels of oxidative stress compared to wild type controls (46).

In our study, AUROC analysis demonstrated that FPF intensity and FPF heterogeneity were consistently capable of differentiating between healthy control eyes and diseased eyes, with FPF intensity being relatively more sensitive than FPF heterogeneity. Both FPF intensity and heterogeneity were positively correlated with BCVA, providing objective confirmation to this subjective measure. Diabetic patients exhibited significantly higher FPF intensity and heterogeneity scores compared to age-matched controls. Both PDR and NPDR subjects exhibited FPF intensity and heterogeneity scores that were 2 and 1.4 times higher, respectively, than values found in age-matched controls. Proposed mechanisms by which mitochondrial dysfunction occurs in diabetic retinopathy include chronic hyperglycemia stimulating the production of sorbitol *via* the polyol pathway, advanced glycation end products (AGEs), and other toxic pro-inflammatory metabolites *via* the diacylglycerol protein kinase C (DAG-PKC) pathway (47–49). Additionally, FPF intensity and heterogeneity were elevated in the DR cohort because of the disproportionately high number of DR patients with macular edema, a phenomenon that is implicated in inner blood-retinal barrier breakdown (50–52). Chronic hyperglycemia can result in microangiopathy and vascular leakage, which can progress to macular edema and capillary occlusion, the latter of which can cause retinal ischemia and subsequent neovascularization (53, 54). The disproportionate prevalence of severe NPDR in our DR cohort likely resulted in comparable FPF intensity and heterogeneity values between the PDR and NPDR groups.

The CSR cohort also exhibited FPF intensity values that were 1.7 times as high as the age-matched control group. While the pathophysiology of CSR is poorly understood, pathological changes are most often located in the macula and result in extravascular fluid leakage through the RPE into the subretinal space (30, 55–57). Daruich et al. was able to demonstrate increased levels of the oxidative stress biomarker, malondialdehyde, in human tears of patients with CSR (58). In our patients, neurosensory or RPE sensory detachments hindered our ability to distinguish the contribution of the disease process on levels of metabolic stress from stress caused by secondary detachments. This is consistent with Elner et al.’s findings which demonstrated FPF elevation in subjects with bilateral CSR who lack subretinal or sub-RPE fluid (14). He hypothesized that elevated FPF levels revealed some ongoing metabolic stress in eyes with the disease even in its sub-clinical presentation.

The RVO group exhibited a similarly elevated mean FPF intensity value to FPF scores found in the CSR cohort, suggesting that comparable levels of oxidative stress could be found in the two conditions. These findings are consistent with Chen et al.’s study, which found elevated levels of oxidative stress in RVO subjects as measured by malondialdehyde 8-hydroxy-2-deoxyguanosine (MDA) and hydrogen peroxide (H_2_O_2_) (59). Retinal venous occlusive disease causes blockage of retinal veins that drain into the central retinal vein, resulting in macular edema, hemorrhaging, and rapid loss of visual acuity from ischemia (60). Importantly, retinal vein occlusion can cause retinal ganglion cell apoptosis under ischemic conditions that generate reactive oxygen species and membrane lipid peroxidation (11). FPF intensity scores were also similar between CRVO and BRVO subjects. FPF heterogeneity, however, was not statistically significantly different between CRVO, BRVO, and age-matched controls. Elevated FPF heterogeneity is hypothesized to reveal variation of FPF signal intensity due to the impact of the disease process on different retinal cells in the macula (4). FPF heterogeneity exhibited lower levels of intra-session repeatability with a coefficient of variability of 16.39% suggesting that FPF heterogeneity may be a more complex indicator of metabolic stress which remains to better understood.

To date, this is the first study to evaluate the validity and reliability of FPF in cohorts of patients with RVO, DR, exudative AMD, and CSR in comparison to control eyes with no evidence of ocular pathology. This is also the first study to investigate retinal FPF in subjects affected by RVO. In 2012, Field et al. used a second generation OcuMet Beacon^®^ device to non-invasively measure elevations in retinal FPF in six patients with non-exudative age-related macular degeneration, three of whom exhibited geographic atrophy (GA), compared to age-matched controls (20). Elner et al. compared data from 14 diabetic patients, 1 advanced non-exudative AMD patients, 1 CSR patients, and 1 retinitis pigmentosa patients and concluded that FPF values were all significantly elevated compared to control subjects (14). Most recently, Chen et al. compared a cohort of 151 control subjects with 117 diabetic patients and found a statistically significant difference in FPF intensity and heterogeneity between the two groups (4). Interestingly, in contrast to our findings, the authors report that higher FPF heterogeneity, rather than FPF intensity, is predictive of poor visual acuity.

It is worth noting some of the limitations in our study design. First, the relatively small sample size of our CSR cohort limits the generalizability of the conclusions. However, this is the first study to include ten CSR subjects in an investigation of retinal flavoprotein autofluorescence; Elner et al.’s study only included 2 CSR subjects and Field et al.’s study compared FPF data from 3 unilateral CSR subjects with their unaffected eyes and 6 age-matched controls (14, 19). The small sample size of our DR cohort restricted our ability to subclassify NPDR patients into early, moderate, and severe stages of clinical severity according to the Early Treatment Diabetic Retinopathy Study (ETDRS) criteria. Small sample size also hindered our ability to stratify RVO cases into ischemic and non-ischemic cases. In the future, studies with larger sample sizes are needed to validate our preliminary results. Second, while we did test for shot-to-shot reproducibility in the form of the intra-session repeatability metric, we did not test longitudinal intersession reproducibility. One FPF image centered at the macula at one time point was included in the analysis for each patient, resulting in an inability to measure the intersession variability of the data. Third, treatment with anti-VEGF injections for some patients included in the DR and exudative AMD cohorts and duration of disease were variables that were not controlled for in this study. Theoretically, earlier therapeutic intervention and administration of anti-VEGF injections on a routine basis could have resulted in better-than-expected FPF and BCVA values. Finally, the median age of the age-matched healthy control subject cohort was within 5 years of all disease groups except for the exudative AMD cohort, which could have potentially confounded the results for this particular disease group. While the OcuMet Beacon^®^ is designed to minimize fluorescence signal from other retinal fluorophores such as lipofuscin that increase with age, it is possible for the retinal fluorescence signal to be partially contaminated by fluorescence emitted by other ocular structures, like the crystalline lens. The spectral curves of tryptophan and non-tryptophan fluorophores in the lens fall between 295 – 329 nm, 364-472 nm, 437-523 nm, respectively, exhibiting minimal overlap with the excitation and emission peaks of retinal flavoproteins (4). It has been previously demonstrated that natural fluorophores in a patient’s lens increase with age, which could potentially affect the fluorescence signal from the retina (18). It is unlikely that the retinal fluorescence signal was contaminated by corneal fluorophores, which exhibit excitation peaks near 300-360 nm and 370-440 nm (4).

In summary, this study highlights the utility of flavoprotein fluorescence as a rapid, noninvasive, and quantitative indicator of mitochondrial dysfunction due oxidative stress in the human retina *in vivo*. FPF intensity was demonstrated to be a robust and sensitive metric for evaluating disease severity in diseased eyes and was significantly correlated with BCVA. Our findings were in agreement with reports from previous studies that FPF intensity has clinically useful diagnostic capability to differentiate healthy eyes from diseased eyes, particularly in exudative AMD subjects. Larger, longitudinal future investigations will be necessary to evaluate its utility in monitoring therapeutic interventions in diseased eyes or predicting pre-structural trajectory of various diseases.

## Data availability statement

The raw data supporting the conclusions of this article will be made available with written request by the authors, without undue reservation.

## Ethics statement

The studies involving human participants were reviewed and approved by New York Eye and Ear Infirmary Institutional Review Board. The patients/participants provided their written informed consent to participate in this study.

## Author contributions

All authors attest that they meet the current ICMJE criteria for Authorship. SA: Conceptualization, Investigation, Methodology, Data Collection, Data Analysis, Writing, Funding acquisition. HA: Writing, Data Analysis, and Interpretation. OO-M: Conceptualization, Data Analysis and Interpretation. JM: Data Collection, Writing. DZ: Writing – Review and editing. CR: Data Analysis and Interpretation, Writing – Review and editing. RR: Conceptualization, Writing – Review and editing, Funding acquisition. All authors contributed to the article and approved the submitted version.

## Supplementary material

Supplementary Figure 1 Box and whisker plots of Flavoprotein Fluorescence (FPF) Heterogeneity between **(A)** unaffected age-matched control subjects compared to RVO, CSR, DR, and Exudative AMD subjects; **(B)** Age-matched controls compared to PDR and NPDR subjects; **(C)** Age-matched controls compared to active and chronic inactive CSR subjects; and **(D)** Age-matched controls compared to CRVO and BRVO subjects. Significant *p* values are shown; all other pairwise comparisons were not statistically significant (*p* > 0.05). RVO, retinal vein occlusion; DR, diabetic retinopathy; wet AMD, wet age-related macular degeneration; CSR, central serous retinopathy; PDR, proliferative diabetic retinopathy; NPDR, non-proliferative diabetic retinopathy; CRVO, central retinal vein occlusion; BRVO, branch retinal vein occlusion.

Supplementary Figure 2 Box and whisker plots of best corrected visual acuity (BCVA) between **(A)** unaffected age-matched control subjects compared to RVO, CSR, DR, and exudative AMD subjects; **(B)** Age-matched controls compared to PDR and NPDR subjects; **(C)** Age-matched controls compared to active and chronic inactive CSR subjects; and **(D)** Age-matched controls compared to CRVO and BRVO subjects. Significant *p* values are shown; all other pairwise comparisons were not statistically significant (*p* > 0.05). RVO, retinal vein occlusion; DR, diabetic retinopathy; CSR, central serous retinopathy; PDR, proliferative diabetic retinopathy; NPDR, non-proliferative diabetic retinopathy; CRVO, central retinal vein occlusion; BRVO, branch retinal vein occlusion.

Supplementary Table 1 Study Population Baseline Characteristics and Demographics (*N = 88*)

Supplementary Table 2 Mean values ± SD and 95% confidence intervals (CI) for FPF Intensity, FPF Heterogeneity, and BCVA in all disease groups compared to unaffected age-matched controls. *Indicates statistical significance.

Supplementary Table 3
**P** values from *post hoc* pairwise comparisons after Kruskal-Wallis Tests comparing FPF Intensity, FPF Heterogeneity, and BCVA Between Disease Groups. ^*^Indicates statistical significance.

Supplementary Table 4
**P** values from *post hoc* pairwise comparisons after Kruskal-Wallis Tests comparing FPF Intensity, FPF Heterogeneity, and BCVA Between PDR, NPDR, and Control Groups. ^*^Indicates statistical significance.

Supplementary Table 5
**P** values from Kruskal-Wallis Tests Comparing FPF Intensity and BCVA Between Active CSR, Chronic Inactive CSR, and Control Groups. Kruskal Wallis post pairwise comparisons indicated that there was no statistically significant difference in FPF heterogeneity between Active CSR, Chronic Inactive CSR, and age-matched controls (*P* = 0.117). ^*^Indicates statistical significance.

Supplementary Table 6
**P** values from Kruskal-Wallis Tests Comparing FPF Intensity, FPF Heterogeneity, and BCVA Between CRVO, BRVO, and Control Groups. FPF heterogeneity between age-matched controls, CRVO, and BRVO subjects were not significant (*P* = 0.112). ^*^Indicates statistical significance.
