# Supplementary figures and images for: Flavoprotein fluorescence elevation is a marker of mitochondrial oxidative stress in patients with retinal disease
Source: Front Ophthalmol (Lausanne). 2023 Feb 16;3:1110501. doi: 10.3389/fopht.2023.1110501 (PMC11182218; doi:10.3389/fopht.2023.1110501)

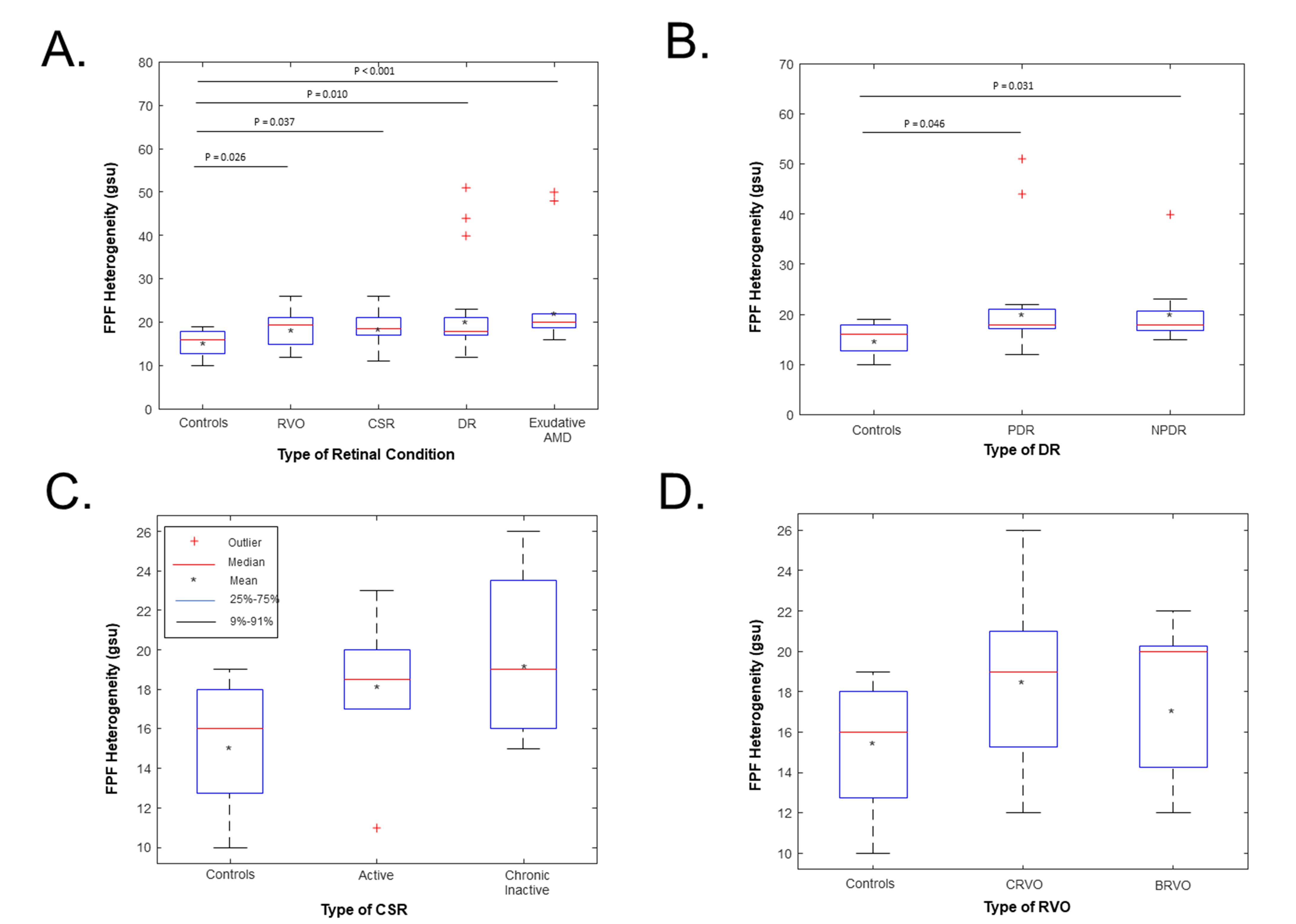

Supplement: Supplementary Figure 1 — Box and whisker plots of Flavoprotein Fluorescence (FPF) Heterogeneity between (A) unaffected age-matched control subjects compared to RVO, CSR, DR, and Exudative AMD subjects; (B) Age-matched controls compared to PDR and NPDR subjects; (C) Age-matched controls compared to active and chronic inactive CSR subjects; and (D) Age-matched controls compared to CRVO and BRVO subjects. Significant p values are shown; all other pairwise comparisons were not statistically significant (p > 0.05). RVO, retinal vein occlusion; DR, diabetic retinopathy; wet AMD, wet age-related macular degeneration; CSR, central serous retinopathy; PDR, proliferative diabetic retinopathy; NPDR, non-proliferative diabetic retinopathy; CRVO, central retinal vein occlusion; BRVO, branch retinal vein occlusion. [file Image_1.tif]

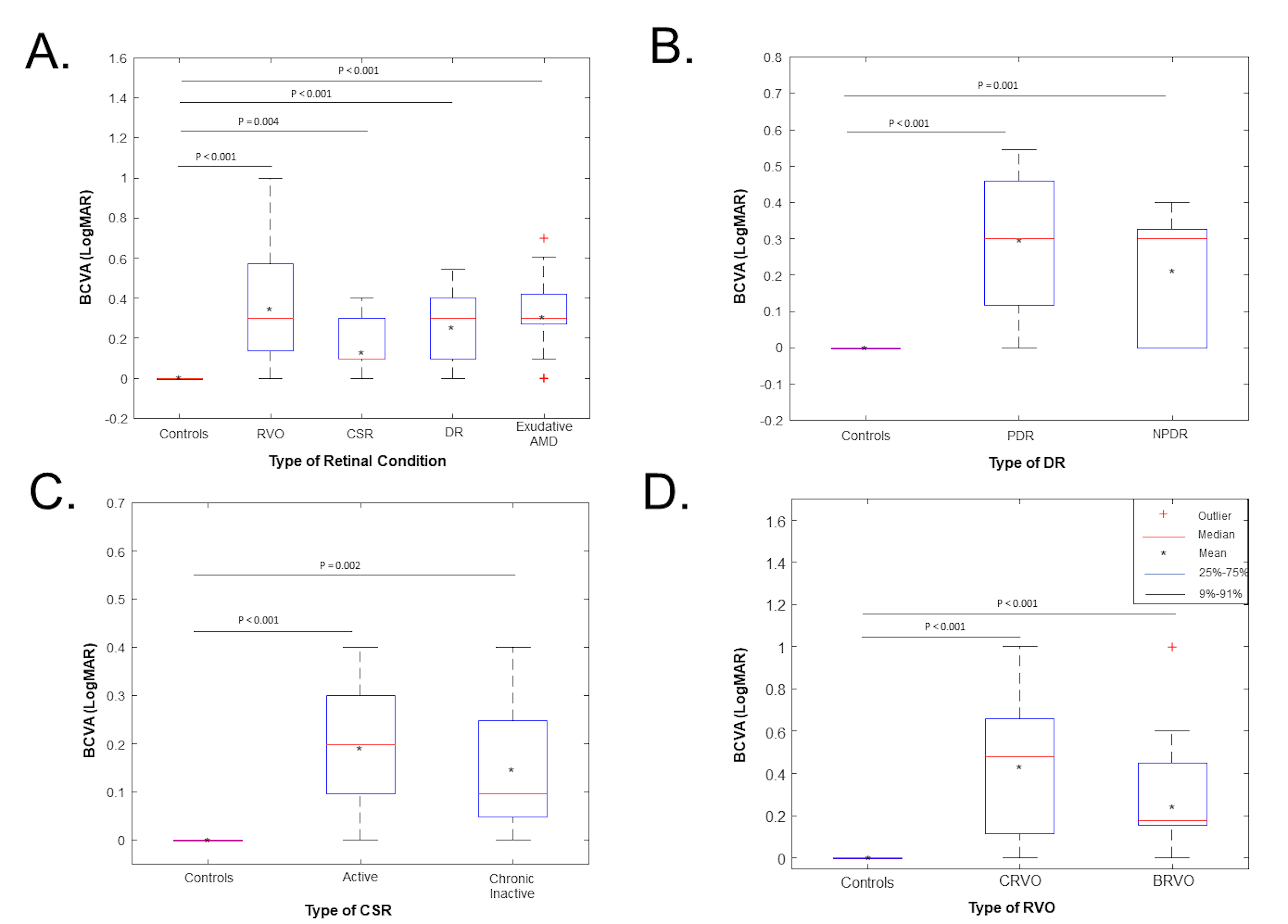

Supplement: Supplementary Figure 2 — Box and whisker plots of best corrected visual acuity (BCVA) between (A) unaffected age-matched control subjects compared to RVO, CSR, DR, and exudative AMD subjects; (B) Age-matched controls compared to PDR and NPDR subjects; (C) Age-matched controls compared to active and chronic inactive CSR subjects; and (D) Age-matched controls compared to CRVO and BRVO subjects. Significant p values are shown; all other pairwise comparisons were not statistically significant (p > 0.05). RVO, retinal vein occlusion; DR, diabetic retinopathy; CSR, central serous retinopathy; PDR, proliferative diabetic retinopathy; NPDR, non-proliferative diabetic retinopathy; CRVO, central retinal vein occlusion; BRVO, branch retinal vein occlusion. [file Image_2.tif]
