# Supplementary material for: Flavoprotein fluorescence elevation is a marker of mitochondrial oxidative stress in patients with retinal disease
Source: Front Ophthalmol (Lausanne). 2023 Feb 16;3:1110501. doi: 10.3389/fopht.2023.1110501 (PMC11182218; doi:10.3389/fopht.2023.1110501)
Supplement: Supplementary Table 1 — Study Population Baseline Characteristics and Demographics (N = 88) [file Table_1.docx]

**Supplementary Table 1. Study Population Baseline Characteristics and Demographics (*N = 88*)**

| **Characteristic** | **Result**** |
| --- | --- |
| ***Unaffected controls:***  Eyes included for analysis:  Age: Median ± interquartile range (IQR) – years  Sex: female, n (%)  Race*: n (%)   - Caucasian - African American - Asian - Hispanic   OD: n (%) | 21  21  55 ± 9  10 (47.6)  10 (47.6)  2 (9.5)  3 (13.3)  6 (28.6)  13 (61.9) |
| ***RVO Subjects:***  Eyes included for analysis:  Age: Median ± interquartile range (IQR) – years  Sex: female, n (%)  Race*: n (%)   - Caucasian - African American - Asian - Hispanic   Subtype: n (%)   - CRVO - BRVO   OD: n (%) | 20  20  59 ± 10  12 (60)  7 (35)  8 (40)  1 (5)  4 (20)  11 (55)  9 (45)  10 (50) |
| ***DR Subjects:***  Eyes included for analysis:  Age: Median ± interquartile range (IQR) – years  Sex: female, n (%)  Race*: n (%)   - Caucasian - African American - Asian - Hispanic   Subtype: n (%)  NPDR  PDR  OD: n (%) | 20  20  59 ± 11.25  8 (40)  3 (15)  6 (30)  3 (15)  8 (40)  9 (45)  11 (55)  11 (55) |
| ***Exudative AMD Subjects:***  Eyes included for analysis:  Age: Median ± interquartile range (IQR) – years  Sex: female, n (%)  Race*: n (%)   - Caucasian - African American - Asian - Hispanic   OD: n (%) | 17  17             70 ± 15  8 (47.1)  14 (82.3)  1 (5.9)  1 (5.9)  1 (5.9)  5 (29.4) |
| ***CSR Subjects:***  Eyes included for analysis:  Age: Median ± interquartile range (IQR) – years  Sex: female, n (%)  Race*: n (%)   - Caucasian - African American - Asian - Hispanic   Subtype: n (%)  Active  Chronic Inactive  OD: n (%) | 10  10  54 ± 11.75  4 (40)  6 (60)  0 (0)  0 (0)  4 (40)  6 (60)  4 (40)  4 (40) |
| Continuous variables are described as Median ± IQR. Categorical variables are described as frequency (percentage).  * Race was reported by the investigators.  ** Percentages may not add up to 100 because of rounding.  ¶  DR denotes diabetic retinopathy, NPDR nonproliferative diabetic retinopathy, PDR proliferative diabetic retinopathy, CSR central serous retinopathy, AMD age-related macular degeneration, RVO retinal vein occlusion, CRVO central retinal vein occlusion, BRVO branch retinal vein occlusion. | |
