# Supplementary material for: Flavoprotein fluorescence elevation is a marker of mitochondrial oxidative stress in patients with retinal disease
Source: Front Ophthalmol (Lausanne). 2023 Feb 16;3:1110501. doi: 10.3389/fopht.2023.1110501 (PMC11182218; doi:10.3389/fopht.2023.1110501)
Supplement: Supplementary Table 2 — Mean values ± SD and 95% confidence intervals (CI) for FPF Intensity, FPF Heterogeneity, and BCVA in all disease groups compared to unaffected age-matched controls. *Indicates statistical significance. [file Table_2.docx]

| **Group** | **FPF intensity mean ± SD** | **FPF intensity 95% CI** | **FPF heterogeneity mean ± SD** | | **FPF heterogeneity 95% CI** | **BCVA in LogMAR mean ± SD** | **BCVA in LogMAR 95% CI** |
| --- | --- | --- | --- | --- | --- | --- | --- |
| Age-Matched Controls | 30.62 ± 8.03 | 26.96 – 34.28 | | 15.62 ± 2.87 | 14.31 - 16.93 | 0.00 ± 0.00 | -- |
|  |  |  | |  |  |  |  |
| All RVO | 53.80 ± 17.97 | 45.39 - 62.21 | | 18.00 ± 4.10 | 16.08 - 19.92 | 0.38 ± 0.32 | 0.23 - 0.53 |
|  |  |  | |  |  |  |  |
| CRVO | 53.55 ± 21.99 | 38.77 – 68.32 | | 18.27 ± 4.47 | 15.27 – 21.28 | 0.43 ± 0.33 | 0.21 – 0.65 |
|  |  |  | |  |  |  |  |
| ­­BRVO | 54.11 ± 12.76 | 44.30 – 63.92 | | 17.67 ± 3.84 | 14.71 – 20.62 | 0.32 ± 0.31 | 0.08 – 0.56 |
|  |  |  | |  |  |  |  |
| All Diabetic Retinopathy | 61.75 ± 19.84 | 52.47 - 71.03 | | 21.80± 10.44 | 16.91 - 26.69 | 0.25 ± 0.18 | 0.17 – 0.34 |
|  |  |  | |  |  |  |  |
| PDR | 61.73 ± 21.76 | 47.10 – 76.35 | | 22.73 ±12.60 | 14.26 – 31.19 | 0.29 ± 0.19 | 0.17 – 0.42 |
|  |  |  | |  |  |  |  |
| NPDR | 61.78 ± 18.51 | 47.55 – 76.00 | | 20.67 ± 7.62 | 14.81 – 26.52 | 0.21 ± 0.17 | 0.08 - 0.34 |
|  |  |  | |  |  |  |  |
| Exudative AMD | 67.47 ± 17.77 | 58.33 - 76.61 | | 23.12 ± 9.91 | 18.02 - 28.21 | 0.32 ± 0.19 | 0.23 - 0.42 |
|  |  |  | |  |  |  |  |
| All CSR | 53.80 ± 14.34 | 43.54 - 64.06 | | 18.70 ± 4.19 | 15.70 - 21.70 | 0.18 ± 0.15 | 0.06 – 0.29 |
|  |  |  | |  |  |  |  |
| Active CSR | 60.00 ± 12.18 | 47.22 – 72.78 | | 18.00 ± 4.00 | 13.80 – 22.20 | 0.20 ± 0.15 | 0.04 – 0.36 |
|  |  |  | |  |  |  |  |
| Chronic Inactive CSR | 44.50 ± 13.39 | 23.29 – 65.71 | | 19.75 ± 4.86 | 12.02 – 27.48 | 0.15 ± 0.17 | -0.12 – 0.42 |
|  |  |  | |  |  |  |  |
| *P* Value | ***1.06 x 10^-8^**** |  | | ***0.002**** |  | ***5.54 x 10^-8^**** |  |

**Supplementary Table 2.** Mean values ± SD and 95% confidence intervals (CI) for FPF Intensity, FPF Heterogeneity, and BCVA in all disease groups compared to unaffected age-matched controls. *Indicates statistical significance.
