# Supplementary material for: Flavoprotein fluorescence elevation is a marker of mitochondrial oxidative stress in patients with retinal disease
Source: Front Ophthalmol (Lausanne). 2023 Feb 16;3:1110501. doi: 10.3389/fopht.2023.1110501 (PMC11182218; doi:10.3389/fopht.2023.1110501)
Supplement: Supplementary Table 3 — P values from post hoc pairwise comparisons after Kruskal-Wallis Tests comparing FPF Intensity, FPF Heterogeneity, and BCVA Between Disease Groups. *Indicates statistical significance. [file Table_3.docx]

**Supplementary Table 3.** *P* values from post hoc pairwise comparisons after Kruskal-Wallis Tests comparing FPF Intensity, FPF Heterogeneity, and BCVA Between Disease Groups. ^*^Indicates statistical significance.

| **FPF Intensity** |  |  |  |  |
| --- | --- | --- | --- | --- |
|  |  |  |  |  |
|  | **Age-Matched Controls** | **RVO** | **CSR** | **DR** |
|  |  |  |  |  |
|  |  |  |  |  |
| **RVO** | **<0.001*** | -- | -- | -- |
|  |  |  |  |  |
| **CSR** | **0.001*** | 0.907 | -- | -- |
|  |  |  |  |  |
| **DR** | **<0.001*** | 0.238 | 0.397 | -- |
|  |  |  |  |  |
| **Exudative AMD** | **<0.001*** | **0.048*** | 0.128 | 0.399 |
|  |  |  |  |  |
| **FPF Heterogeneity** |  |  |  |  |
|  |  |  |  |  |
|  | **Age-Matched Controls** | **RVO** | **CSR** | **DR** |
|  |  |  |  |  |
| **RVO** | **0.026*** | -- | -- | -- |
|  |  |  |  |  |
| **CSR** | **0.037*** | 0.790 | -- | -- |
|  |  |  |  |  |
| **DR** | **0.010*** | 0.744 | 1.000 | -- |
|  |  |  |  |  |
| **Exudative AMD** | **<0.001*** | 0.055 | 0.184 | 0.108 |
|  |  |  |  |  |
| **BCVA** |  |  |  |  |
|  |  |  |  |  |
|  |  |  |  |  |
|  | **Age-Matched Controls** | **RVO** | **CSR** | **DR** |
|  |  |  |  |  |
|  |  |  |  |  |
| **RVO** | **<0.001*** | -- | -- | -- |
|  |  |  |  |  |
| **CSR** | **0.004*** | 0.119 | -- | -- |
|  |  |  |  |  |
| **DR** | **<0.001*** | 0.305 | 0.471 | -- |
|  |  |  |  |  |
| **Exudative AMD** | **<0.001*** | 0.947 | 0.144 | 0.359 |
