# Supplementary material for: Flavoprotein fluorescence elevation is a marker of mitochondrial oxidative stress in patients with retinal disease
Source: Front Ophthalmol (Lausanne). 2023 Feb 16;3:1110501. doi: 10.3389/fopht.2023.1110501 (PMC11182218; doi:10.3389/fopht.2023.1110501)
Supplement: Supplementary Table 4 — P values from post hoc pairwise comparisons after Kruskal-Wallis Tests comparing FPF Intensity, FPF Heterogeneity, and BCVA Between PDR, NPDR, and Control Groups. *Indicates statistical significance. [file Table_4.docx]

| **FPF Intensity** | |  | |  | |
| --- | --- | --- | --- | --- | --- |
|  | |  | |  | |
|  | | **Age-Matched Controls** | | **PDR** | |
|  | |  | |  | |
|  | |  | |  | |
| **PDR** | | **< 0.001*** | | -- | |
|  | |  | |  | |
| **NPDR** | | **< 0.001*** | | 0.948 | |
|  | |  | |  | |
| **FPF Heterogeneity** | |  | |  | |
|  | | **Age-Matched Controls** | | **PDR** | |
| **PDR** | | **0.046*** | |  | |
| **NPDR** | | **0.031*** | | 0.788 | |
| **BCVA** | |  | |  | |
|  | |  | |  | |
|  | |  | |  | |
|  | | **Age-Matched Controls** | | **PDR** | |
|  | |  | |  | |
|  | |  | |  | |
| **PDR** | | **< 0.001*** | | -- | |
|  | |  | |  | |
| **NPDR** | | **0.001*** | | 0.274 | |
|  |  | |  | |  |

**Supplementary Table 4.** *P* values from post hoc pairwise comparisons after Kruskal-Wallis Tests comparing FPF Intensity, FPF Heterogeneity, and BCVA Between PDR, NPDR, and Control Groups. ^*^Indicates statistical significance.
