# Supplementary material for: Flavoprotein fluorescence elevation is a marker of mitochondrial oxidative stress in patients with retinal disease
Source: Front Ophthalmol (Lausanne). 2023 Feb 16;3:1110501. doi: 10.3389/fopht.2023.1110501 (PMC11182218; doi:10.3389/fopht.2023.1110501)
Supplement: Supplementary Table 5 — P values from Kruskal-Wallis Tests Comparing FPF Intensity and BCVA Between Active CSR, Chronic Inactive CSR, and Control Groups. Kruskal Wallis post pairwise comparisons indicated that there was no statistically significant difference in FPF heterogeneity between Active CSR, Chronic Inactive CSR, and age-matched controls (P = 0.117). *Indicates statistical significance. [file Table_5.docx]

**Supplementary Table 5.** *P* values from Kruskal-Wallis Tests Comparing FPF Intensity and BCVA Between Active CSR, Chronic Inactive CSR, and Control Groups.  Kruskal Wallis post pairwise comparisons indicated that there was no statistically significant difference in FPF heterogeneity between Active CSR, Chronic Inactive CSR, and age-matched controls (*P* = 0.117). ^*^Indicates statistical significance.

| **FPF Intensity** |  |  |
| --- | --- | --- |
|  |  |  |
|  | **Age-Matched Controls** | **Active CSR** |
|  |  |  |
|  |  |  |
| **Active CSR** | **< 0.001*** | -- |
|  |  |  |
| **Chronic Inactive CSR** | 0.074 | 0.229 |
|  |  |  |
| **BCVA** |  |  |
|  |  |  |
|  |  |  |
|  | **Age-Matched Controls** | **Active CSR** |
|  |  |  |
|  |  |  |
| **Active CSR** | **< 0.001*** | -- |
|  |  |  |
| **Chronic Inactive CSR** | **0.002*** | 0.775 |
|  |  |  |
