# Supplementary material for: Flavoprotein fluorescence elevation is a marker of mitochondrial oxidative stress in patients with retinal disease
Source: Front Ophthalmol (Lausanne). 2023 Feb 16;3:1110501. doi: 10.3389/fopht.2023.1110501 (PMC11182218; doi:10.3389/fopht.2023.1110501)
Supplement: Supplementary Table 6 — P values from Kruskal-Wallis Tests Comparing FPF Intensity, FPF Heterogeneity, and BCVA Between CRVO, BRVO, and Control Groups. FPF heterogeneity between age-matched controls, CRVO, and BRVO subjects were not significant (P = 0.112). *Indicates statistical significance. [file Table_6.docx]

**Supplementary Table 6.** *P* values from Kruskal-Wallis Tests Comparing FPF Intensity, FPF Heterogeneity, and BCVA Between CRVO, BRVO, and Control Groups. FPF heterogeneity between age-matched controls, CRVO, and BRVO subjects were not significant (*P* = 0.112). ^*^Indicates statistical significance.

| **FPF Intensity** |  |  |
| --- | --- | --- |
|  |  |  |
|  | **Age-Matched Controls** | **CRVO** |
|  |  |  |
|  |  |  |
| **CRVO** | **< 0.001*** | -- |
|  |  |  |
| **BRVO** | **< 0.001*** | 0.619 |
|  |  |  |
| **BCVA** |  |  |
|  |  |  |
|  |  |  |
|  | **Age-Matched Controls** | **CRVO** |
|  |  |  |
|  |  |  |
| **CRVO** | **< 0.001*** | -- |
|  |  |  |
| **BRVO** | **< 0.001*** | 0.708 |
|  |  |  |
